# Supplementary figures and images for: Association of bariatric surgery with all-cause mortality and incidence of obesity-related disease at a population level: A systematic review and meta-analysis
Source: PLoS Med. 2020 Jul 28;17(7):e1003206. doi: 10.1371/journal.pmed.1003206 (PMC7386646; doi:10.1371/journal.pmed.1003206)

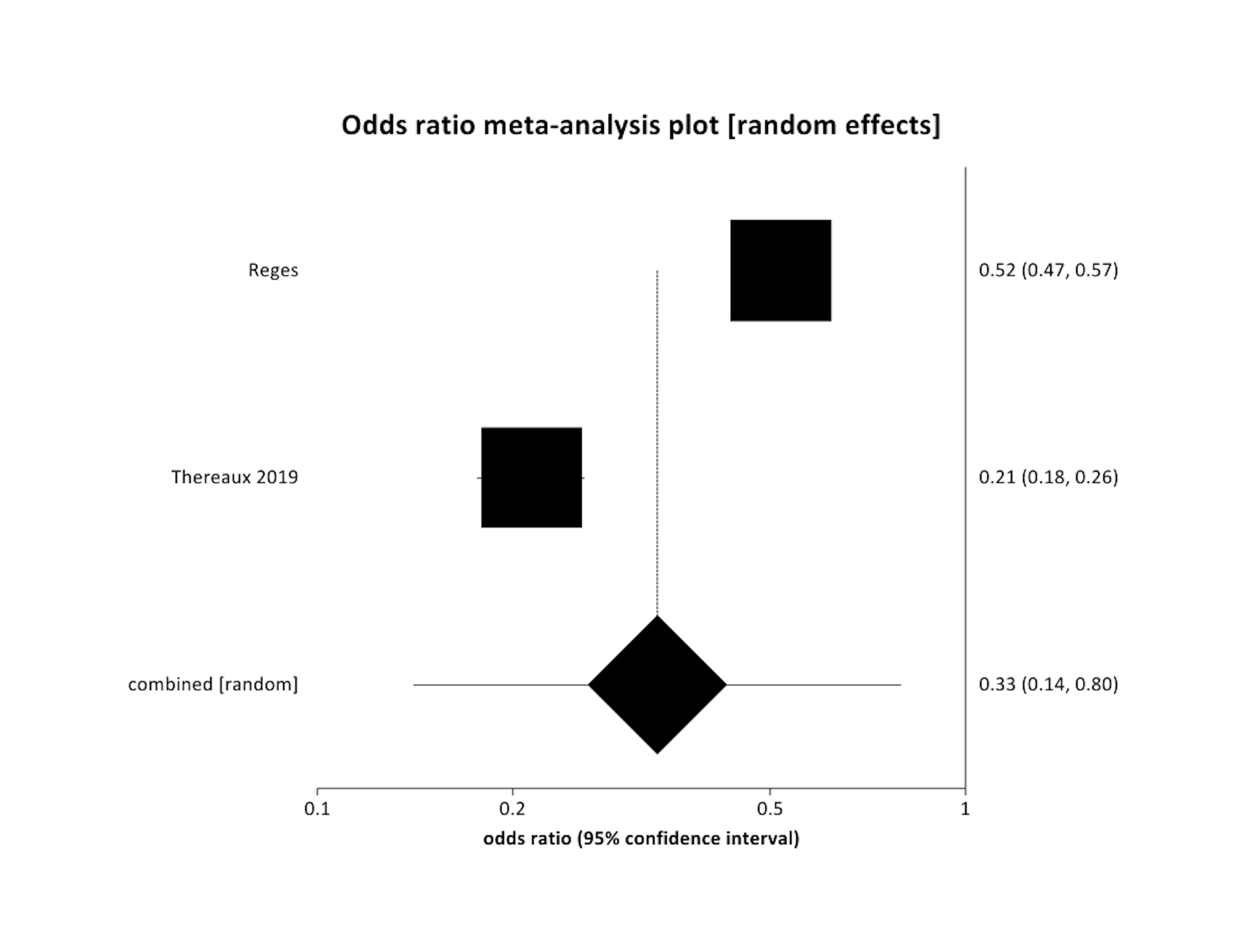

Supplement: S1 Fig — (TIF) [file pmed.1003206.s001.tif]

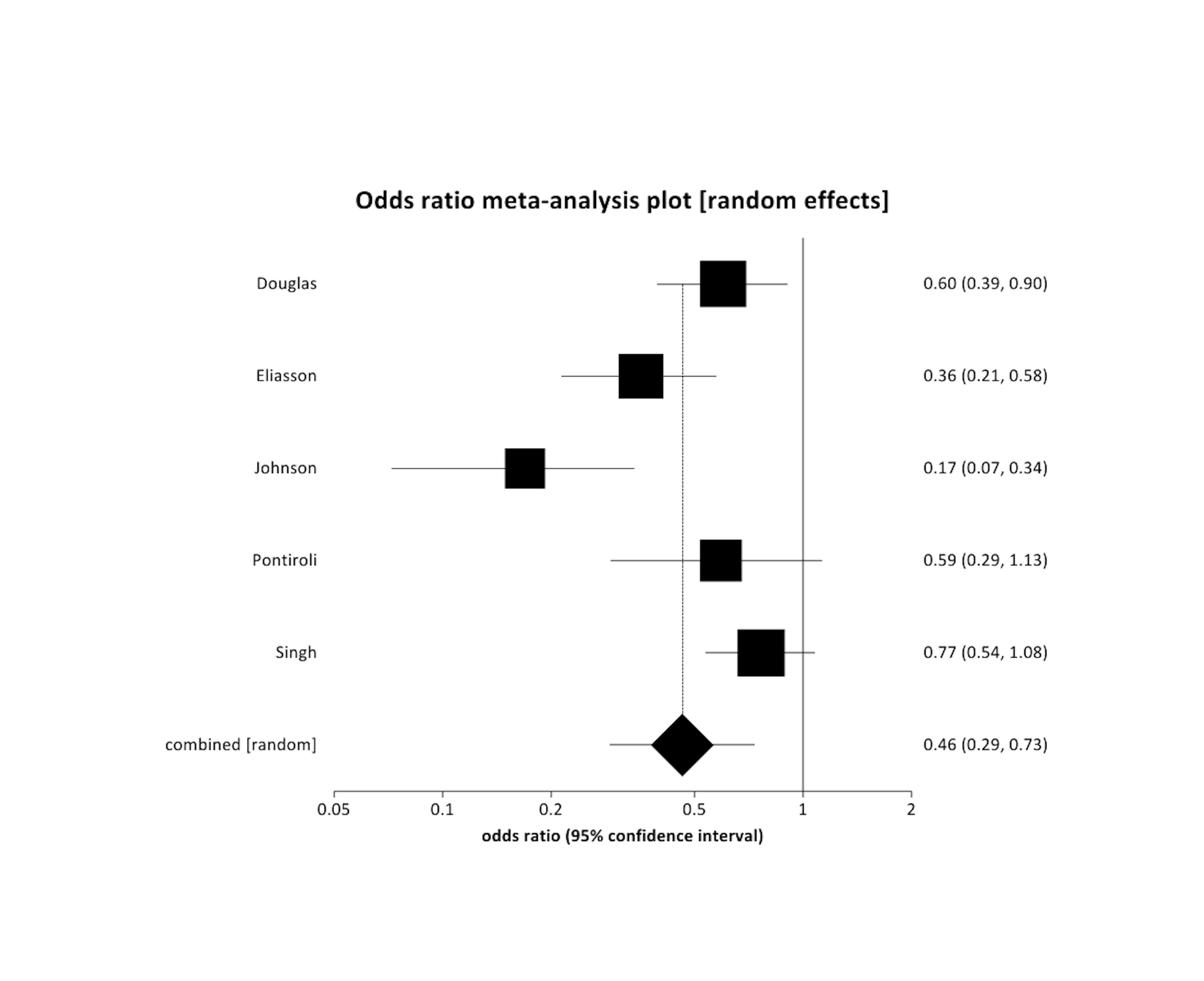

Supplement: S2 Fig — (TIF) [file pmed.1003206.s002.tif]
