# Supplementary material for: Association of bariatric surgery with all-cause mortality and incidence of obesity-related disease at a population level: A systematic review and meta-analysis
Source: PLoS Med. 2020 Jul 28;17(7):e1003206. doi: 10.1371/journal.pmed.1003206 (PMC7386646; doi:10.1371/journal.pmed.1003206)
Supplement: S1 Table — BPD, biliopancreatic diversion; DS, duodenal switch; VBG, vertical banded gastroplasty. (DOCX) [file pmed.1003206.s003.docx]

*S1 table:* Details of form of bariatric surgery performed within each study (VBG – vertical banded gastroplasty, BPD – biliopancreatic diversion, DS – duodenal switch).

| **Author** | **Procedure Type** | | | | |
| --- | --- | --- | --- | --- | --- |
|  | Gastric Bypass | Sleeve | Gastric Band | VBG | BPD/DS |
| Arterburn[25] | 1844 | 381 | 249 | 8 | 18 |
| Backman[26] | 18418 | - | - | - | - |
| Bailly[35] | 27313 | 45698 | 29616 | - | - |
| Ceriani[36] | - | - | - | - | 472 |
| Douglas[37] | 1421 | 613 | 1829 | 4 | 4 |
| Eliasson[38] | 6132 | - | - | - | - |
| Flum[39] | 3328 | - | - | - | - |
| Johnson[40] | - | - | - | - | - |
| Kauppila[41] | 36678 | - | 5450 | 5487 | - |
| Moussa[42] | - | - | - | - | - |
| Moussa[27] | 1543 | 585 | 1480 | 4 | 24 |
| Perry[28] | 7977 | - | - | 216 | - |
| Persson[29] | 20680 | - | 772 | 678 | 165 |
| Pontiroli[30] | - | - | 385 | - | - |
| Singh[31] | 2010 | 1158 | 1965 | - | 37 |
| Reges[32] | 1388 | 3362 | 3635 | - | - |
| Thereaux 2018[33] | 4331 | 3445 | 7592 | - | 282 |
| Thereaux 2019[34] | 4525 | 3674 | - | - | - |
