# Supplementary material for: Association of bariatric surgery with all-cause mortality and incidence of obesity-related disease at a population level: A systematic review and meta-analysis
Source: PLoS Med. 2020 Jul 28;17(7):e1003206. doi: 10.1371/journal.pmed.1003206 (PMC7386646; doi:10.1371/journal.pmed.1003206)
Supplement: S2 Table — (DOCX) [file pmed.1003206.s004.docx]

*S2 table:* Newcastle-Ottawa Score for all included studies

| **Author** | **Selection (Maximum 4 stars)** | **Comparability (Maximum 2 stars)** | **Exposure (maximum 3 stars)** |
| --- | --- | --- | --- |
| Arterburn[25] | ★★★★ | ★★ | ★★★ |
| Backman[26] | ★★★ | ★ | ★★★ |
| Bailly[35] | ★★★★ | ★★ | ★★★ |
| Ceriani[36] | ★★★★ | ★★ | ★★★ |
| Douglas[37] | ★★★★ | ★★ | ★★★ |
| Eliasson[38] | ★★★★ | ★★ | ★★★ |
| Flum[39] | ★★★★ | ★ | ★★★ |
| Johnson[40] | ★★★★ | ★ | ★★★ |
| Kauppila[41] | ★★★★ | ★ | ★★★ |
| Moussa[42] | ★★★★ | ★ | ★★★ |
| Moussa[27] | ★★★★ | ★★ | ★★★ |
| Perry[28] | ★★★★ | ★ | ★★★ |
| Persson[29] | ★★★★ | ★ | ★★★ |
| Pontiroli[30] | ★★★★ | ★★ | ★★★ |
| Singh[31] | ★★★★ | ★★ | ★★★ |
| Reges[32] | ★★★★ | ★★ | ★★★ |
| Thereaux 2018[33] | ★★★★ | ★★ | ★★★ |
| Thereaux 2019[34] | ★★★★ | ★★ | ★★★ |
