# Supplementary material for: Association of bariatric surgery with all-cause mortality and incidence of obesity-related disease at a population level: A systematic review and meta-analysis
Source: PLoS Med. 2020 Jul 28;17(7):e1003206. doi: 10.1371/journal.pmed.1003206 (PMC7386646; doi:10.1371/journal.pmed.1003206)
Supplement: S3 Table — Data in parentheses represent 95% confidence interval. T2DM, type 2 diabetes; VTE, venous thromboembolism. (DOCX) [file pmed.1003206.s005.docx]

*S3 table: Analysis of development of incident comorbid disease via adjusted odds ratio data. Data in brackets represents 95% confidence interval;* T2DM – type 2 diabetes; VTE – venous thromboembolism.

| **Author** | **T2DM** | **Hypertension** | **Obstructive Sleep Apnea** | **Dyslipidemia** | **Ischemic Heart Disease** | **Cardiac failure** | **VTE** |
| --- | --- | --- | --- | --- | --- | --- | --- |
| Arterburn[25] | - | - | - | - | - | - | - |
| Backman[26] | 0.77 (0.67-0.89) | - | - | - | - | - | - |
| Bailly[35] | 0.18 (0.17-0.19) | - | - | - | - | - | - |
| Ceriani[36] | - | - | - | - | - | - | - |
| Douglas[37] | 0.68 (0.55-0.83) | 0.35 (0.27-0.45) | 0.55 (0.37-0.82) | - | 0.59 (0.40-0.87) | - | - |
| Eliasson[38] | - | - | - | - | 0.51 (0.29-0.91) | - | - |
| Flum[39] | - | - | - | - | - | - | - |
| Johnson[40] | - | - | - | - | - | - | - |
| Kauppila[41] | - | - | - | - | - | - | - |
| Moussa[42] | - | - | - | - | - | - | - |
| Moussa[27] | - | - | - | - | - | - | 0.52 (0.35-0.78) |
| Perry[28] | - | - | - | - | - | - | - |
| Persson[29] | - | - | - | - | - | 0.37 (0.29-0.46) | - |
| Pontiroli[30] | - | - | - | - | - | - | - |
| Singh[31] | - | 0.41 (0.34-0.50) | - | - | 0.85 (0.61-1.19) | 0.57 (0.34-0.96) | - |
| Reges[32] | - | - | - | - | - | - | - |
| Thereaux 2018[33] | 0.06 (0.04-0.09) | - | - | - | - | - | - |
| Thereaux 2019[34] | - | 0.22 (0.18-0.26( | - | 0.12 (0.09-0.15) | - | - | - |
| **Pooled odds ratio** | **0.28 (0.11-0.73)** | **0.32 (0.21-0.47)** | **-** | **-** | **0.67 (0.49-0.90)** | **0.43 (0.29-0.64)** | **-** |
