# Supplementary material for: Association of bariatric surgery with all-cause mortality and incidence of obesity-related disease at a population level: A systematic review and meta-analysis
Source: PLoS Med. 2020 Jul 28;17(7):e1003206. doi: 10.1371/journal.pmed.1003206 (PMC7386646; doi:10.1371/journal.pmed.1003206)
Supplement: S1 Text — (DOCX) [file pmed.1003206.s006.docx]

*S1 Text: Additional information for literature search:*

Detailed search terms utilized for Medline database:

("Bariatric Surgery"[MeSH – Medical Subject Heading] OR bariatric surgery) AND (mortality OR survival OR diabetes OR metabolic OR hypertension OR sleep apnoea OR cardiac OR angina OR heart disease OR myocardial infarction OR dyslipidaemia OR dyslipidemia OR thromboembolism) AND (national OR registry OR population)

Entry terms included within "Bariatric Surgery" Medical Subject Heading (MeSH):

- Surgeries, Bariatric
- Surgery, Bariatric
- Metabolic Surgery
- Metabolic Surgeries
- Surgeries, Metabolic
- Surgery, Metabolic
- Bariatric Surgical Procedures
- Procedure, Bariatric Surgical
- Procedures, Bariatric Surgical
- Surgical Procedure, Bariatric
- Surgical Procedures, Bariatric
- Bariatric Surgeries
- Stomach Stapling
- Stapling, Stomach
