# Supplementary material for: Association of bariatric surgery with all-cause mortality and incidence of obesity-related disease at a population level: A systematic review and meta-analysis
Source: PLoS Med. 2020 Jul 28;17(7):e1003206. doi: 10.1371/journal.pmed.1003206 (PMC7386646; doi:10.1371/journal.pmed.1003206)
Supplement: Attachment — Submitted filename: Response to Reviewers.docx [file pmed.1003206.s008.docx]

**Response to Reviewers**

Requests from the editors:

*Not a request but just to note that this paper was submitted, and is being considered in PLOS Medicine as part of a special issue focussed on the Determinants, Consequences and Management of Obesity.

This has been noted by the authors.

*The authors have appropriately reported the work as per PRISMA guidelines for systematic reviews, but would also be good to state (although this is not compulsory for the journal) whether the protocol for the systematic review was established and either published or registered before the start of the SR conduct - ie, either as a published protocol paper or in the PROSPERO registry (<https://www.crd.york.ac.uk/PROSPERO/>)

This study was not prospectively registered and this has been stated on Page 7 line 132-133.

*At this stage, we ask that you include a short, non-technical Author Summary of your research to make findings accessible to a wide audience that includes both scientists and non-scientists. The Author Summary should immediately follow the Abstract in your revised manuscript. This text is subject to editorial change and should be distinct from the scientific abstract. Please see our author guidelines for more information: <https://journals.plos.org/plosmedicine/s/revising-your-manuscript#loc-author-summary>

This has been included (Page 4, line 71-82)

*In the last sentence of the Abstract Methods and Findings section, please describe the main limitation(s) of the study's methodology. One thing to note here (see later) is that the systematic review did not, as far as this reader can tell, aim to assess adverse effects associated with bariatric surgery, only improvements on mortality and obesity-related disease.

This has now been added (Page 2 line 57-61). The abstract is now 377 words in length but remains below the 500 word maximum word limit specified in the instructions for authors.

*If possible, please change the referencing format to the PLOS Medicine style using square brackets to enclose sequential numbered callouts in the text (rather than superscript numbers) - this should be easy if referencing software was used.

This has been changed to PLOS Medicine style.

*Main text covering study limitations might also include a point about not covering adverse effects in this review.

This has now been included (Page 15 line 347 to 353).

-----------------------------------------------------------

Comments from the reviewers:

Reviewer #1:

In this meta-analysis, the methods are correct, and the conclusions are sound.

There are a few details that require attention:

1.     under strategy, there is no mention of paper search in PubMed

Pubmed was included within the search of Medline but this has now been specifically stated (Page 7 line 123).

2.     figures should report weight of studies

The statistical package that was utilised to produce these figures (StatsDirect) does not specifically report values within the figures for study weighting but this is graphically represented within the Forest Plots by the size of square for each of the individual studies.

3.     the analysis would be more satisfactory if reporting surgeries separately; only if no difference appears, then the surgeries can be considered together.

Unfortunately due to the nature of reported data it was not possible to perform a specific sub-group analysis by procedure type and this has been included within the limitations of the study (Page 14 line 346-347). However the overall aim was to assess the association of bariatric surgery with long-term mortality and incidence of new onset obesity-related disease at a national level, which this study still fulfils.

Reviewer #2: Alex McConnachie, Statistical Review

Wiggins et al present a systematic review and meta-analysis of whole-population studies using administrative datasets, of the association between bariatric surgery and adverse outcomes, confirming the beneficial effects seen in randomised trials in general population settings. This review considers the statistical elements of the paper.

First, to be pedantic, the word "effect" in the title of the paper should perhaps be changed - since this meta-analysis is combining observational studies, the associations observed cannot be interpreted as causal effects, even if few would dispute that they are.

This has been changed to the word ‘association’ as suggested

As a whole, the statistical methods used, their presentation, and interpretation are good. My comments are therefore relatively minor.

The literature search was reportedly carried out in January 2020. I assume that is when it was last updated?

The wording of this section has been changed to clarify that the literature search was last updated in January 2020 (Page 7, line 128 to 129).

In the final sentence of the first paragraph of the methods, the words "formal" and "formally" should maybe be removed. I am not sure what it would mean to say this was done "informally", so better to simply say it was not done.

This has been altered as suggested (Page 7 line 132-133).

In the final paragraph of the methods, covering the statistical methods, the word "significant" can be removed (twice). I do not think it adds anything.

This has been altered as suggested (Page 8 line 166 and 171).

For Egger's test, I have read that it is not recommended with fewer than 10 studies. This is only a rule of thumb, so the results can still be reported, but it is perhaps a limitation.

This is a fair comment however in our experience this recommendation is not an absolute rule. So we have chosen to still report the results of the test, but state in the limitations that due to the limited number of studies the outcome of bias from the Egger tests should be cautiously interpreted [Page 15 line 353-358].

For those outcomes where there was only one study, so that a pooled analysis could not be performed, it would help the reader if the result of that one trial were shown in the text.

This data has been added to the manuscript (Page 10 line 218 to 220 and Page 11 line 242 to 244).

One phrase that crops up throughout the paper is that included studies should include an appropriate control group. It was not clear to me what was meant by "appropriate" here. Perhaps this could be expanded upon.

This has been clarified within the text (Page 7 line 140-141).

Also, it is stated that whilst mortality was analysed in terms of hazard ratios, the obesity related disease incidence data were analysed as event rates in each group. Did any studies present adjusted odds ratios, or use propensity scores, or other methods to control for confounding? Would it have been better to perform the meta-analysis using these adjusted ORs, rather than the raw event counts? Not performed due to discrepancies in data available and limited number of studies report adjusted OR’s for these outcomes. Need to review and add this data.

**RESPONSE:**

Although data regarding adjusted ORs was available from some studies this method of reporting was not included in each of the individual studies included within the analysis. Therefore, on this basis the examination of incidence of obesity related disease was performed relative to event rates rather than the reported adjusted OR’s. However, where this data was available this analysis has been performed and show consistency the results of the event rate analysis and this has been included in the results section (Page 11 line 246 to 252) and added as supplementary table 3 (Page 33).

-----------------------------------------------------------

Reviewer #3:
*    Is the manuscript well organized and written clearly enough to be accessible to non-specialists?
Yes, this an interesting and well written manuscript.

*    What are the main claims of the paper and how significant are they for the discipline?
The authors claim:
1.    In the abstract (line 55-57) that "This pooled analysis has demonstrated that bariatric surgery can improve long-term all-cause mortality and reduce the incidence of obesity-related disease in patients with obesity for the whole operated population."and that "The results suggest that broader access to bariatric surgery for people with obesity may reduce the long-term sequelae of this disease and provide population-level benefits"

2.    In the discussion: "This meta-analysis of large-scale registry studies indicate that patients receiving bariatric surgery have improved long-term mortality rates compared to controls at a population level."

3.    In the introduction: "bariatric surgery improves all-cause mortality" (line 34 abstract), "reduces overall risk of developing multiple cancer types" (line 73), "previous pooled analyses have demonstrated that bariatric surgery reduces long-term all-cause mortality" (line86-87). These causal claims about previous research are not supported by any randomised controlled study or clinical study with an appropriate control group. What about SOS?

Data from the Swedish Obese Subjects (SOS) study has now been referenced as part of these statements to provide further support to these statements (Page 5 line 98-101). Results from the SOS study have also been further expanded upon within the discussion (Page 16 line 375-381).

These main claims may be important for the discipline, but they are generally too optimistic by indicating causality (e.g. by the inappropriate use of the term "effect of") several places in the title, abstract, and manuscript.  Importantly, all studies included in the meta-analysis were observational and none could infer causality, only associations, as mentioned in the title of several of the included studies (particularly those published in JAMA). A meta-analysis of "association studies" does not increase the evidence level to infer causation. In view of this, my advice to the authors is to remove or rephrase all undocumented causal claims in the manuscript.

These sections have been rephrased throughout the manuscript as suggested (Title of manuscript; page 2 line 37; Page 11 line 236; Page 12 line 260; Page 12 line 269; Page 14 line 321).

*    Are the claims properly placed in the context of the previous literature? Have the authors treated the literature fairly?
Yes

*    Do the data and analyses fully support the claims? If not, what other evidence is required?
The claims in the first part of the discussion (lines 209-210, and 218) are partly supported by the data: "bariatric surgery is associated with reduced all-cause long-term mortality" and "reduced incidence of ", but whether the studies had "appropriate control groups" is discussable (see below).
Tthe claims of the beneficial effects (indicating causal effects) of bariatric surgery on all-cause mortality and cardiovascular mortality are overly optimistic and should be modified. Most importantly, the term "Effect" in the title "Effect of bariatric surgery.." should be removed, and may be substituted with "Associations of bariatric surgery …" as in the JAMA-papers referred to. The term "effect" should also be replaced in line 36, because the design of the current meta-analysis is not appropriate to assess any cause-effect relationship.

These issues have been addressed as detailed above.

Regarding other outcomes (e.g. obesity related diseases), the findings are in accordance with previously published randomised and non-randomised controlled studies.
In my view, the main problem with the majority (if not all) of studies included in the meta-analysis is the absence of an appropriate control group, which the authors stated that they aimed to include in the methods sections (line 42 and line 112). Although this limitation is partly addressed in the discussion (lines 263-266), several other limitations with the control groups regarding imbalances in important known and unknown confounding factors in general, and in the included studies in particular, need to be addressed.

Please see section below for responses to these individual issues.

First, as mentioned by the authors, less than 1% of eligible patients receive bariatric surgery. In general, and as shown in the paper by Reges et al in JAMA (ref 29), these patients probably have higher socioeconomic status (higher education and income) than controls not receiving bariatric surgery. It is well known that socioeconomic status is an important predictor of all-cause mortality. Another example is the higher prevalence of people with
schizophrenia in the control group in the study by Arterburn et al. (ref 22) etc.

These factors have now both been addressed within the limitations section (page 13 line 314 to Page 14 line 320).

Second, few, if any (?) control groups consisted of treatment seeking patients with obesity who received an adequate non-surgical treatment for obesity? An ethical committee would find it very difficult to accept a control group not receiving any kind of active treatment in a clinical trial, and comparing treatment-groups with no-treatment groups will probably favor the treatment group. FYI, our group has tried to partly overcome this bias by comparing treatment seeking patients with obesity who opted for either bariatric surgery or tertiary care based conservative treatment in an observational cohort study (Jakobsen GS, et al. Association of Bariatric Surgery vs Medical Obesity Treatment With Long-term Medical Complications and Obesity-Related Comorbidities. JAMA 2018;319(3):291-301) regarding remission and incidence of obesity related diseases.

A further statement regarding the suitability of the control groups utilized has been added to the limitations section and the suggested paper by Jakobsen et al. has been references within the text as presenting a potential model of utilizing patients within a specialised medical programme for treatment of obesity as a more suitable control cohort in future studies. (Page 14 line 326-343).

Third, several unmeasured confounders in the various studies might have biased the results.

More detail has been provided regarding potential confounders which may have caused bias to the results (Page 13 line 314 to Page 14 line 320). A further analysis using adjusted OR for the development of comorbid disease has now also been completed to partially account for these potential confounders as suggested by the statistical reviewer 2 (Page 11 line 246 to 252) and in supplementary table 3 (Page 34).

In addition, although beyond the scope of the article, the well-known detrimental long-term effects of bariatric surgery should at least be addressed in the discussion.

We have included a discussion regarding potential complications of bariatric surgery (Page 15 line 347 to 353).

-----------------------------------------------------------

Reviewer #4: Dear Editor,
Thank you for the opportunity to review the manuscript entitled: " Effects of bariatric surgery on all-cause mortality and incidence of obesity-related disease at a population level - systematic review and meta-analysis".

In the manuscript, the authors address the impact of bariatric surgery on all-cause mortality and new onset of metabolic comorbidities in a systematic review of studies published between 2000 and January, 2020.

My general impression of the study is that it is overall well conducted and the manuscript well written. The topic of the study, as well as the conclusion, are already known and accepted by bariatric surgeons and physicians with positive attitudes towards bariatric surgery. Despite the evidence supporting bariatric surgery for many patients with morbid obesity, the results still have a hard time reaching a wide acceptance. Thus, there is a need for further well written papers summarizing the beneficial effects (as well as side-effects and complications) of bariatric surgery, and these papers need to reach a wide audience of readers, such as through PLoS Medicine. Furthermore, the present study included only population-based studies, which is a novel approach.

While I have a few (mostly minor) comments and questions to the authors, it is my opinion that the manuscript should be of interest to a wide readership, and thus should be eligible for publication in PLoS Medicine.

General comments:

My main overall concern with the manuscript is related to the transparency of the presentation, and to some extent to the limitations of the studies included.

First, while all search terms are presented in the first section of the methods section, the description remains unclear in the sense that there are too may possible options for the search terms. For optimal transparency the reader should be able to redo the literature search, and end-up with similar results. I assume that the authors clustered search terms related to study group (i.e. bariatric, obesity, bariatric surgery etc.), outcome, and study level separately. If the authors could present the search-terms more specifically, it would be helpful.

This has been addressed and a more specific search methodology has been included in the manuscript (Page 7 line 124 to 127).

I fully agree with the authors that avoiding randomized trials, in particular single-center studies, can be supported by the tendency of these studies to overestimate treatment effects (and the sometimes low external validity). However, I don't fully understand why matched, prospective, multicenter studies (such as the SOS-trial) were not included in the study. Could the authors specify this decision better?

The rationale for including only population based studies has been explained further within the manuscript (Page 7 line 147 to page 8 152). These studies were specifically selected to ensure that results could be generalizable to the population of patients receiving bariatric surgery rather than being limited to those receiving surgery in highly specialized units participating in research studies to demonstrate the external validity of potential benefits of bariatric surgery at a population level.

Perhaps some of these issues has been presented already in a study plan, published and registered elsewhere, but I can't find it. Was the systemic review registered before the study was conducted? Perhaps this could be specified with registration number if it was, or with a short comment if not.

This study was not prospectively registered and this has been stated on Page 7 line 132-133.

Finally, it is likely that intervention groups, in general, are better controlled for comorbid disease, thus identifying more cases with disease and thereby influencing the risk for new onset of disease. Thus, there is a risk that the meta-analyses in the study overestimates the potential beneficial effect on new onset of disease. Providing remission of disease could have added to the overall view of the effect of bariatric surgery on metabolic comorbidities, but the authors have provided a reasonable explanation as to why they did not choose to include this outcome. However, a potential differential bias exists and should be presented in the limitation section.

The potential influence of these factors to introduce bias has now been specifically mentioned within the limitation section (Page 14, line 326 to 343). A further analysis using adjusted OR for the development of comorbid disease has now also been completed to partially account for these potential confounders (Page 11 line 246 to 252) and in supplementary table 3 (Page 33).


Minor additional comments:

Abstract: Information on eligibility criteria is incomplete, and should preferably be extended in the methods section. Furthermore, a short section on the major limitations of the study could be informative.

These recommendations have been added to the abstract (Page 2 line 42 to 45 and Page 2 line 57 to 61).

Methods section: Did the authors contact authors from the included studies to obtain or confirm specific data from the included studies? This could have been helpful in order to have more detailed data. For instance, by contacting specific authors, surgical methods known to have inferior results (i.e. the vertical banded gastroplasty, as well as the unknown /miscellaneous group) could have been excluded. It is not a major issue though, since keeping these methods in the study is likely to underestimate the treatment effects of the intervention.

Authors of individual included studies were not directly contacted to obtain more detailed data and a statement regarding this has been added to the methods section (Page 8 line 158 to 159).

Supplementary Table 1: The studies could be more uniformly presented, preferably with author name and reference number (as raised numbers)

This has been corrected.
